# The influence of social status and network structure on consensus building in collaboration networks

**DOI:** 10.1007/s13278-016-0389-y

**Published:** 2016-09-15

**Authors:** Ilire Hasani-Mavriqi, Florian Geigl, Subhash Chandra Pujari, Elisabeth Lex, Denis Helic

**Affiliations:** Knowledge Technologies Institute, KTI, Graz University of Technology, Inffeldgasse 13/VI, Graz, 8010 Austria

**Keywords:** Opinion dynamics, Consensus building, Collaboration networks, Naming Game

## Abstract

In this paper, we analyze the influence of social status on opinion dynamics and consensus building in collaboration networks. To that end, we simulate the diffusion of opinions in empirical networks and take into account both the network structure and the individual differences of people reflected through their social status. For our simulations, we adapt a well-known Naming Game model and extend it with the *Probabilistic Meeting Rule* to account for the social status of individuals participating in a meeting. This mechanism is sufficiently flexible and allows us to model various society forms in collaboration networks, as well as the emergence or disappearance of social classes. In particular, we are interested in the way how these society forms facilitate opinion diffusion. Our experimental findings reveal that (i) opinion dynamics in collaboration networks is indeed affected by the individuals’ social status and (ii) this effect is intricate and non-obvious. Our results suggest that in most of the networks the social status favors consensus building. However, relying on it too strongly can also slow down the opinion diffusion, indicating that there is a specific setting for an optimal benefit of social status on the consensus building. On the other hand, in networks where status does not correlate with degree or in networks with a positive degree assortativity consensus is always reached quickly regardless of the status.

## Introduction

It is our natural predisposition to interact with people who have a high social status in our social communities. Customarily, our social interactions and, to some extent, our behavior are influenced by actions of individuals with a high social status. In the field of social psychology, the social status theory attempts to explain this phenomenon (Markovsky et al. 1993; Walker et al. 2000; Willer 1999). According to it, people tend to form their connections in a social network to maximize their perceived social benefits arising from the social status of their connections. Also, in the work of Guha et al. (2004) the authors relate social status to the mechanism of link formation in a social network, hypothesizing that people with a lower social status are more likely to create (directed) links with people of a higher social status.

In this paper, however, we are not interested in the relation between the social status and the process of link formation, but rather in the relation between social status and *dynamical processes* that may take place in a social or collaboration network (i.e., a special case of social network, in which users collaborate). One example of such dynamical process is a so-called opinion dynamics process. In our daily lives, we interact with our peers, discuss certain problems, exchange opinions and try to reach some kind of consensus. The question we want to answer in this paper is how social status influences such processes in a collaboration network. For example, in a university class there is a lively discussion between a student and her mentor regarding their newest experimental results and their interpretation. The mentor has a higher social status than the student, due to a superior education, a broader experience and a higher position in the organizational hierarchy. Undoubtedly, while trying to reach a consensus, the student will be influenced by opinions of her mentor because of the latter’s convincing power (Castellano et al. 2009; Latané 1981). The literature (Castellano et al. 2009) identifies this process as dynamics of agreement/disagreement between persons belonging to a social group. For clarity, in this paper we will refer to it as opinion dynamics.

### Problem

The aim of this work is to extend our previous investigations (Hasani-Mavriqi et al. 2015) in respect of the influence of social status on the process of reaching consensus within a social community that has a heterogeneous distribution of social status, by studying the underlying network structure. In particular, we investigate new empirical networks and construct synthetic networks to analyze the impact of degree assortativity and the correlation between degree and social status on opinion dynamics. While there is a substantial body of work on opinion dynamics (see Sect. 6) in general settings, we focus on a more specific and more realistic situation in which the dynamics are influenced not only by the network structure and the relevant parameters but also by the intrinsic properties of every single node in the network, such as social status. In other words, we study the interplay between structure, dynamics and exogenous node characteristics and how these complex interactions influence the process of consensus building.

### Approach and methods

In the field of statistical physics (Castellano et al. 2009), opinion dynamics is commonly studied by applying mathematical models and analytic approaches. To make these complex problems tractable for mathematical analysis, researchers make simplifications, such as presenting opinions as sets of numbers, ignoring the network structure (a typical approach from e.g., mean-field theory) and neglecting the individual differences between nodes. Simplifications narrow the scope of research down to theoretical models, which typically do not consider empirical data. Even so, statistical physics constitutes important basics for the state-of-the-art research on social dynamics in collaboration networks. In this paper, we build upon these basics.

We take a computational approach and analyze opinion dynamics by simulating the diffusion of opinions in empirical collaboration networks (specifically, we study datasets from a Q&A site StackExchange and a co-authorship dataset). In our simulations, we consider the network structure, apply a set of simple rules for opinion diffusion and take into account people’s individual differences (e.g., their social status). In particular, we simulate scenarios of peer interactions in empirical datasets assuming that the status theory holds and observe the consequences. We model the dynamics of opinion spreading by adapting a well-known *Naming Game* model (Baronchelli et al. 2006b) and extending it by incorporating a mechanism to configure the degree of the influence of social status on the network dynamics. We termed this mechanism the *Probabilistic Meeting Rule*. Through parametrization, we are able to explore various scenarios from the opposite sides of the spectrum: (i) We can completely neglect the status by allowing any two individuals to exchange their opinions regardless of their social status (an *egalitarian* society) (Arneson 2013); (ii) we can have opinions flowing only in one direction—from individuals with a higher social status to those with a lower social status (a *stratified* society) (Weber 1964); (iii) we can probabilistically model any situation in between these two extreme cases, that is, a case in which opinions are very likely to flow from individuals with a higher social status to those with a lower social status, but with small probability they can also flow into the other direction (a *ranked* society) (Weber 1964).

### Contributions

The main contributions of our work are twofold. Firstly, with our paper we contribute to the field of opinion dynamics *methodologically*. Secondly, with our work we also make an *empirical* contribution.

Our methodological contribution can be summarized as follows. To model various scenarios of how social status may influence the opinion dynamics, we have invented the Probabilistic Meeting Rule (see Sect. 2.2) and extended a standard Naming Game model with that rule. The extension is flexible and may reflect a variety of interesting scenarios, such as the emergence or disappearance of social classes in collaboration networks. Further, we provide an initial analysis on how this meeting rule may influence the consensus building process. This analysis allows us to obtain an intuition on the possible outcomes of our simulations. The opinion flow between different user groups can be easily controlled through our computational approach for parameter estimation (see Sect. 2.3). We also analyze the influence of network structure, particularly the influence of degree assortativity, and the correlation between degree and status on the process of consensus reaching in collaboration networks.

From the empirical point of view, we made a much-needed contribution to the limited body of research on Naming Game and empirical data (Gao et al. 2014) and obtained very interesting empirical experimental results. For example, based on the status theory it can be expected that consensus can be reached faster when social status plays a role. However, our results only partially confirm this expectation. In particular, if an opinion flows only in one high- to low-status direction, opinions do not converge at all since there are always a few people who do not adopt the common opinion from the network. However, with only a low influence of social status convergence is reached faster than with no status at all (as in a standard Naming Game). These results suggest that finding the optimal process of consensus reaching is a tuning act of how to integrate social status in the opinion dynamics. In addition, our investigations on the role of network structure on opinion dynamics reveal that (i) hubs are important factors for spreading a single common opinion among other nodes and (ii) in networks with a positive assortativity degree or a degree sequence decorrelated with user’s social status, the consensus is reached without external intervention.

The StackExchange empirical networks used in our previous work (Hasani-Mavriqi et al. 2015) are disassortative networks, i.e., they have a negative degree assortativity coefficient. In disassortative networks, high-degree nodes are on average connected to nodes with low(er) degree (Noldus and Mieghem 2015). In this work, we extend our experiments with an additional type of empirical network, namely assortative networks, in which physical connections between low and high agents are very rare. We turn to co-authorship networks as an example of networks that exhibit a positive degree assortativity coefficient, indicating that, on average, nodes with similar degrees are connected together.

## Methodology

### Naming Game

Naming Game (Baronchelli et al. 2005, 2006a, b; Dall’Asta et al. 2006a, b) is a networked agent-based topology, in which agent-to-agent interactions take place based on predefined gaming rules. In particular, agents exchange their opinions and try to reach a consensus about the name of an unknown object. When all agents in the network agree on the name, the network is considered to have established a common opinion.

**Fig. 1:**
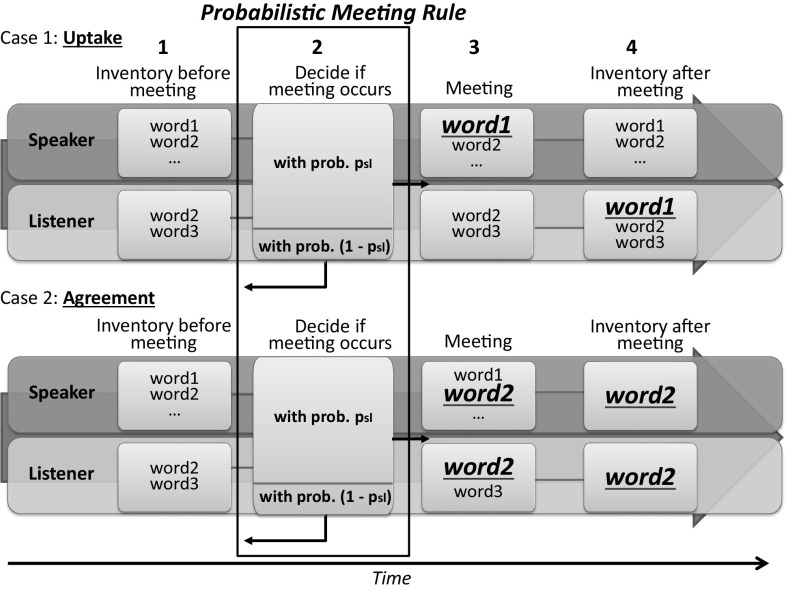
Naming Game meeting. The classical Naming Game consists of *steps 1*, *3* and *4*, whereas our extension also includes the *step 2*. In *step 2*, we decide whether the meeting between two agents occurs by evaluating Probabilistic Meeting Rule (Eq. 1). For illustration, consider a ranked society with stratification factor $\beta =0.0001$. *Example 1*: Speaker’s status $s_s=101$ and listener’s status $s_l=7967$. The meeting probability evaluates to $p_{\text{sl}}=0.45$. We then draw a number from [0, 1] uniformly at random (e.g., 0.93) and compare it with $p_{\text{sl}}$—the meeting does not take place. *Example 2*: Let $s_s=576$ and $s_l=865$, which leads to the meeting probability $p_{\text{sl}}=0.97$. We again draw a random number from [0, 1] (e.g., 0.77)—in this case the meeting takes place. If the meeting takes place, two scenarios are possible. (1) If the speaker transmits a word (*red*) that is unknown by the listener, the listener adds it to her inventory (*uptake*). (2) If the word chosen by the speaker is also known to the listener, they both agree on this word. In this case, they both remove all other words from their inventories and keep only the transmitted one (*agreement*) (color figure online)

Agents in the game are represented as nodes of a network, and edges between two agents allow them to interact with each other. Names are represented with an inventory of words, and each agent has her own inventory to store the words. Technically, an inventory is a set (i.e., a bag) of words. In the initial state, the inventories are empty. Two random adjacent agents are chosen in each simulation step to interact through a meeting: One agent is declared as a speaker and the other as a listener. In the course of the meeting, the speaker selects a word from her inventory and communicates it to the listener (note that if the speaker’s inventory is empty, a new unique word is created and stored in the inventory). After communicating the word to the listener, two scenarios are possible (see Fig. 1):

1. the word is not in the listener’s inventory—the word is added to listener’s inventory,
2. otherwise, both speaker and listener agree on that word and remove all other words from their inventories—they agree on the selected word.

### Naming Game and social status

We modify the Naming Game to account for social status. As before, the agents are represented as network nodes, edges denote whether two agents can interact or not, and names (opinions) are represented as word inventories.

The first difference between our model and a standard Naming Game is the simulation initialization. We initialize the inventories with a given number of selected words from a given vocabulary. The words are selected (with replacement uniformly) at random from the vocabulary. This results in an initial state where each opinion occurs with the same probability.

Secondly, we adopt the social status that governs how agent interactions are turned into meetings—not every agent interaction is turned into a meeting. During each interaction, a random agent and a random neighbor are chosen to have a meeting. Then, the speaker and the listener are assigned randomly. Based on the difference between the speaker’s and the listener’s statuses, we randomly decide whether the meeting occurs.

To decide whether a meeting takes place, we introduce the Probabilistic Meeting Rule. Basically, the Probabilistic Meeting Rule is a function that takes the agents’ social statuses as input and, based on the difference between the speaker’s and listener’s status, calculates the probability of the meeting taking place. The rule is defined by the following equation:

1 $$p_{\text{sl}} = \min \left( 1, {e}^{\beta \cdot (s_s - s_l)}\right),$$

where $s_s$ is the speaker’s status, $s_l$ is the listener’s status and $\beta \ge 0$ is the *stratification factor*. The stratification factor $\beta$, which can be viewed as a measure of conformance to the agent’s social status, is a tuning parameter in our model. The above equation results in the following probabilities. If the speaker’s status is higher than the listener’s status, $p_{\text{sl}}$ has the value of 1, that is, such a meeting always takes a place. If the opposite is true, various scenarios are possible, depending on the value of the stratification factor. For example, $\beta =0$ indicates an *egalitarian* society and $p_{\text{sl}}$ is always equal to 1. However, if we slowly increase the stratification factor, $p_{\text{sl}}$ will start to decay and in general will take a value between 0 and 1, which signifies a *ranked* society (see the running example in Fig. 1). If we continue to increase $\beta$, we will soon (because of the exponential term in the equation) reach a situation where $p_{\text{sl}}$ for all practical matters is equal to 0. In other words, we have reached a *stratified* society where meetings take place only if the speaker’s status is higher than the listener’s status but never in the opposite case.

**Fig. 2:**
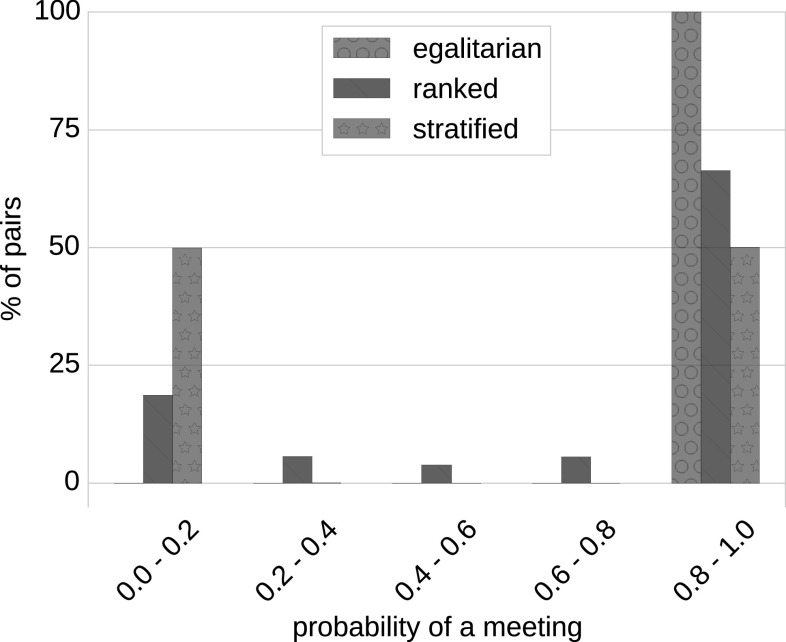
Naming Game and social status. The application of the *Probabilistic Meeting Rule* to our datasets and the emergence of social classes based on the stratification factor $\beta$ are illustrated. The *green bar* with *circle* texture indicates an *egalitarian* society that corresponds to $\beta =0$, in which each agent can meet every other agent. With an increase in $\beta$, our society becomes more conservative (as represented with the *blue bars* with *line* texture) and becomes a *ranked* society. In* red bars* with *circle* texture, we observe a two-class society, that is, a *stratified* society (color figure online)

The application of our Probabilistic Meeting Rule to our datasets is depicted in Fig. 2. The probability of a meeting taking place is shown in correlation with the percentage of pairs of agents participating in that meeting. The above-mentioned scenarios are represented as follows: *egalitarian* society (corresponds to $\beta =0$)—green bar (circle texture), *ranked* society (e.g., $\beta =0.0001$)—blue bar (line texture) and *stratified* society (e.g., $\beta =1$)—red bar (star texture).

### Estimating stratification factor

In this section, our primary goal is to investigate how the stratification factor $\beta$ from Probabilistic Meeting Rule (Eq.1) can be estimated such that the opinion flow between different classes of agents can be easily controlled. We first draw a line in the distribution of agents’ statuses and separate the agents into two classes: *high* (agents with the status above 90th percentile) and *low* (agents below 90th percentile) class. Our focus lies on the estimation of the expected meeting probability between low- and high-status agents. Please note, however, that the methodology presented here can be applied also in a general setting to estimate, for example, expected meeting probability between low-to-low, or high-to-high agents.

The expected meeting probability depends on the differences between agents’ social status, which in turn are random variables with unknown probability density functions. Formally, the problem is to calculate the expectations of a function (Probabilistic Meeting Rule) of a difference of two random variables, which are conditioned on their particular values, that is, they are conditioned on either being a low or a high agent.

Let *X* be a random variable (r.v.) representing a user’s social status. The probability density function (PDF) of the r.v. *X* is given with *p*(*x*). We define now a new random variable conditioned on a specific value of that variable $x_h$, that is, let us consider a random variable *U* for a low-status agent and a random variable *V* for a high-status agent. The PDF of *U* is then given by $p(u) = p(x | x \le x_h)$ and PDF of *V* by $p(v) = p(x | x > x_h)$. Both of these PDFs can be obtained by normalizing with a cumulative and complementary cumulative distribution function evaluated at $x_h$.

To consider the differences between agents’ social statuses, we would need to define a third r.v. $Z = U - V$, and under the assumption that the r.v. *U* and *V* are independent, we could calculate the PDF of *Z* by calculating the convolution integral for *U* and *V*. Finally, we can define the expected value of Probabilistic Meeting Rule ${\text{e}}^{\beta \cdot z}$ as follows:

2 $$E[{\text{e}}^{\beta \cdot z}] = \int _{-\infty }^{\infty } {\text{e}}^{\beta \cdot z} \cdot p(z) {\text{d}}z$$

Since in practice none of these steps is tractable for the analytic solution, we resort to the empirical and approximative parameter estimation. To that end, we first create an empirical distribution for the random variable *Z*. First, we split agents into two classes: low and high defined by, for example, the 90th percentile (although the choice for $x_h$ is in fact arbitrary) in the distribution of agents’ status values. Second, we iterate over all the links in the network and keep only low-to-high pairs to construct an empirical distribution of the differences between agents’ statuses. Please note that the same procedure may be repeated for estimation of, for instance, the expected meeting probability of low-to-low or any other interesting pairs (instead of keeping low-to-high pairs we just need to keep the pairs in question). From this distribution, we then draw a random sample of size *N* and estimate the expectation value for ${\text{e}}^{\beta \cdot z}$ by applying the well-known Monte Carlo estimation (Metropolis and Ulam 1949):

3 $$E[{\text{e}}^{\beta \cdot z}] = \dfrac{1}{N} \sum _{i=1}^{N} {\text{e}}^{\beta \cdot z_i}$$

Our empirical solution is flexible and can be easily adapted to consider opinion flow in other agents’ groups (e.g., high-to-high). By defining the percentage of allowed opinion flow between agents in different groups, we can determine $\beta$ for networks of various structure and scope.

## Datasets and experiments

### Datasets

In our experiments, we use two types of empirical datasets: (i) the first one is derived from a Q&A site (StackExchange^1^) and (ii) the second one is a co-authorship dataset introduced in Tang et al. (2008).

In StackExchange, users collaborate, ask questions and give answers on particular problems. After an iterative discussion process, users exchange their opinions, find solutions to a problem and agree on the best suggested solutions (Tausczik et al. 2014). Such Q&A sites have a reputation system which rewards users via reputation scores based on their contributions (Halavais et al. 2014; Movshovitz-Attias et al. 2013). Based on the policies of this reputation system, users get appropriate reputation scores for giving good answers, asking good questions or voting on questions/answers of other users. It is evident that high-reputation users contribute high-quality answers (Movshovitz-Attias et al. 2013). We expect that high-reputation users also demonstrate high convincing power during the agreement process, influencing opinions of other (low-reputation) users. In our experiments, we apply reputation scores as a proxy for the social status and these two terms are used interchangeably throughout the paper. The StackExchange platform does not indicate associations between users or friendship links. For that reason, we turn our attention to collaboration networks which we extract by analyzing co-posting activities of users in order to have social ties between them (Adamic and Adar 2001; Halavais et al. 2014; Tang et al. 2012). In Q&A sites, a co-posting activity between two users refers to a scenario under which two users comment on the same post. Thus, if two users contributed in any way to a same post, they are connected via an edge in the collaboration network. We analyze the following StackExchange language datasets: French, Spanish, Chinese, Japanese, German and English. They are available for downloading for research purpose from the StackExchange dataset archive.

We constructed our co-authorship network from the empirical dataset presented in Tang et al. (2008) that is freely available under.^2^ In this co-authorship dataset, publication data are combined from three different sources: DBLP, CiteSeer and Google Scholar, and the problem of the author name disambiguation is addressed properly. Two authors are connected via an edge in the co-authorship network if they co-authored at least a publication together. The dataset provides citation counts for each author, which is used in our case as a proxy for author’s reputation.

### Datasets statistics

The details of our empirical networks (derived from the above-mentioned datasets) and their properties are given in Table 1, with the number of nodes (*n*), number of edges (*m*), mean ($\mu$), median ($\mu _{1/2}$), standard deviation ($\sigma$) of the reputation scores, assortativity coefficient (*r*) and modularity (*Q*).

**Table 1:** StackExchange and co-authorship datasets

Dataset	Type	n	m	*μ*	*μ*_1/2_	*σ*	*r*	*Q*
StackExch.	French	1478	6668	298	111	1273	−0.23	0.31
	Spanish	1584	6908	196	101	554	−0.19	0.38
	Chinese	1985	8556	160	61	477	−0.15	0.41
	Japanese	2069	11,155	328	77	1535	−0.16	0.32
	German	2316	12,825	285	103	1219	−0.16	0.32
	English	30,656	192, 983	199	48	1654	−0.19	0.33
Co-auth.	AMiner	1,057,194	3,634,124	20	2	138	0.15	0.67

Description of StackExchange and co-authorship datasets with the number of nodes (*n*), number of edges (*m*), mean ($\mu$), median ($\mu _{1/2}$) and standard deviation ($\sigma$) of the reputation scores, assortativity coefficient (*r*) and modularity (*Q*)

Among our StackExchange datasets, the English network is the largest one with 30,656 nodes and 192,983 edges, whereas the French is the smallest one with 1478 nodes and 6668 edges in the network. The German, Japanese, Chinese and Spanish networks lie in between the English and French networks in terms of network size. The co-authorship dataset is much larger in size compared with all StackExchange datasets; with 1,057,194 nodes and 3,634,124 edges, it constitutes the largest dataset in our experiments.

The negative assortativity coefficient *r* in our StackExchange datasets indicates a negative correlation (Newman 2003) between reputation scores over the network edges. In other words, users with lower reputation scores are more likely to connect to users with higher reputation scores. In particular, a typical post in our datasets has many users with low scores (e.g., who post a question) and only a few or even only a single user with a high score (e.g., who answers the question). This finding is in line with the assumptions from the social status theory. The Chinese network has the lowest absolute assortativity coefficient among our networks, indicating that in this network there is a smaller chance of connection with a dissimilar reputation score. The Japanese and French networks have the highest absolute assortativity coefficient. The co-authorship dataset is characterized with a positive assortativity coefficient *r*, which is typical for co-authorship networks in general (Noldus and Mieghem 2015), indicating that, on average, nodes with similar reputation scores are connected together. Particularly, this means that authors having similar social status in their community tend to publish an article together.

The modularity score is a measure of strength of the community structure in a network. A high modularity score indicates the existence of strong communities in the network, while a low modularity score means that the community structure is not that strong (Newman 2006). In our StackExchange networks, we observe low modularity values corresponding to a very weak or almost nonexistent community structure. As previously shown in a network without communities, in general Naming Game converges quickly to a single opinion (Baronchelli et al. 2006b). In contrary, our co-authorship network exhibits much higher modularity value; thus, the community structure in this network is stronger.

**Fig. 3:**
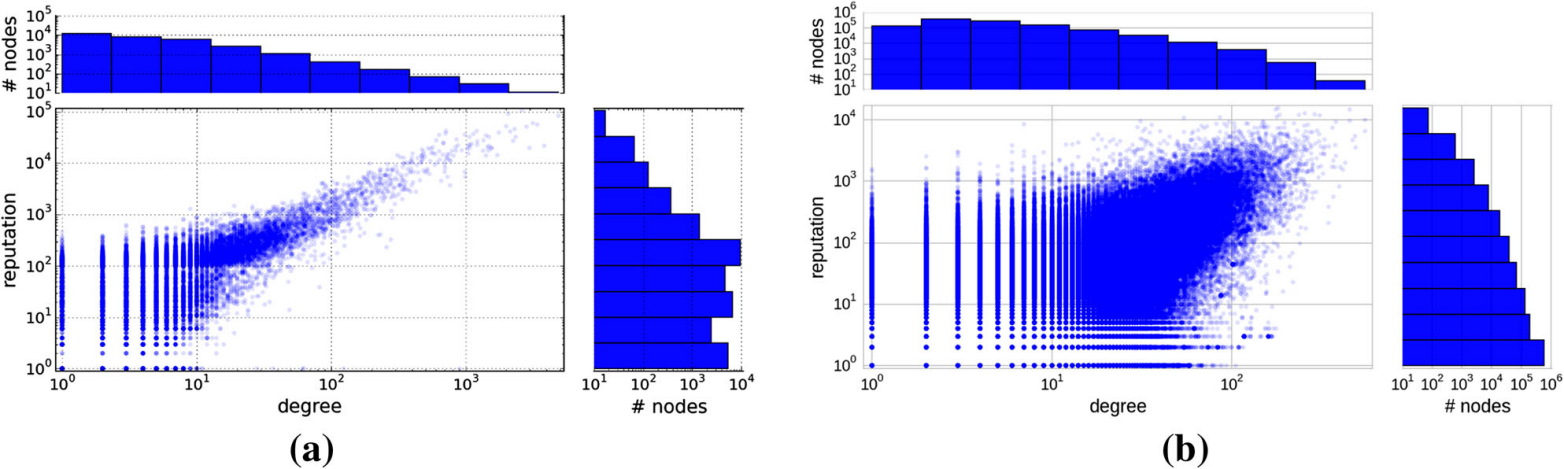
Distribution of reputation scores. Correlation between the distribution of reputation scores and node degrees for the English StackExchange network (**a**) and co-authorship network (**b**). The subplots on the *right* show the heterogenous distribution of reputation scores in the both networks. The subplots on the *top* present the heterogenous distribution of node degrees. In the *middle*, the scatter plot of reputation scores versus node degrees is shown. The Pearson correlation coefficient between degrees and reputation scores is 0.88 for the English StackExchange network. All other StackExchange datasets have comparable distributions and correlation coefficients. In the case of the co-authorship network, the Pearson correlation coefficient between degrees and reputation scores is 0.54. As it can be seen from the plot in **b**, it is evident that some authors with a high citation count have a low degree (i.e., low number of co-authored publications), but there are also cases of authors with a low citation count and a high degree (i.e., they are active in scientific collaboration, but their publications have a low citation count)

The distribution of reputation scores and node degrees resembles a heterogenous distribution for all networks, which indicates that the majority of users in our collaboration networks have low-reputation scores. Figure 3a shows the English StackExchange network, in which the correlation between the reputation scores and the node degrees is a linear correlation with a Pearson correlation coefficient of 0.88. All other StackExchange datasets have comparable properties. In the case of the co-authorship network shown (see Fig. 3b), the Pearson correlation coefficient between the degree and the reputation score is 0.54. It is evident that there are cases of authors having a high citation count (used as a proxy for reputation) but low degree, which indicates that they possess a low number of co-authored publications that are frequently cited. For illustration purposes, we further investigated this property of our co-authorship dataset and retrieved the names of the authors having a low degree (lower than the 90th percentile) and a high citation count (higher than the 90th percentile). For example, the author Dennis M. Volpano^3^ is characterized in our dataset with a degree of 6 and a citation count of 750. After checking the author’s website and digital libraries such as IEEE Xplore, it is obvious that the author published most of his publications as a single author or in collaboration with other few authors, but his publications received a considerable attention from the community and are highly cited. The opposite scenarios are also possible, which correspond to authors being active in scientific collaboration (high degree), but their publications have a low citation count.

### Simulations

In our experiments, we simulate Naming Game extended with the Probabilistic Meeting Rule. The simulation framework is provided as an open source project.^4^ Our experiments consist of the following steps:

1. We calculate the stratification factor $\beta$ using the approach from Sect. 2.3, getting the values for the stratification factor that we need, to reflect a given situation. For all networks, we define five percentages, which correspond to the society forms defined earlier in this paper and control the opinion flow from low- to high-status agents (i.e., 100 %—*egalitarian*, 75, 50 and 25 %—*ranked* and 0 %— *stratified* society).
2. Each agent’s inventory is initialized with a fixed number of three opinions (represented through numbers from 0 to 99). These opinions are selected uniformly at random from a bag of opinions to ensure that each opinion occurs with the same probability.
3. We once create meeting sequences and apply the same sequences for the different values of stratification factors. Initialization of agent inventories differs for each meeting sequence, but same initializations are used for all $\beta$. Hence, it is ensured that the randomness between $\beta$ is insignificant, due to the same meeting sequence and same initialization for different $\beta$.
4. For each meeting sequence, depending on the network size, we define the number of user interactions (iterations) for the simulations. We perform 4 million interactions for the largest StackExchange network (English), 1 million interactions for the five other StackExchange networks and 20 million interactions for the co-authorship network.
5. We run 100 simulations per $\beta$ and report the averaged simulation results to account for statistical fluctuations in the simulations.
6. During the simulations, we store important information such as the appearance of agents as listeners/speakers, their participation in overall interactions versus successful meetings and the evolution of the agent’s inventory size.
7. We modify the initialization of the agents’ inventories to differentiate between opinions assigned to low- and high-status agents, respectively, in order to evaluate the final agreement of agents.

## Results and discussion

**Fig. 4:**
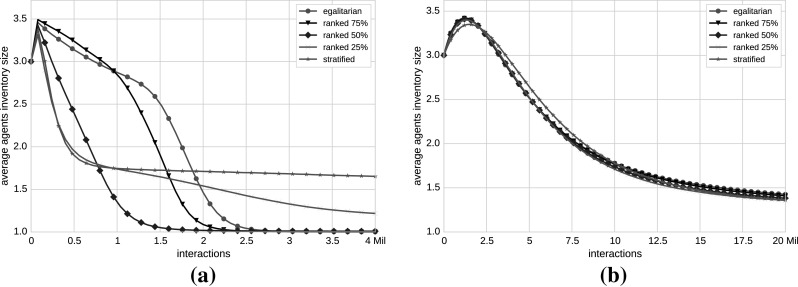
Inventory size evolution averaged over 100 runs per $\beta$. Mean values of the agent’s inventory size in relation to the number of interactions for English StackExchange (**a**) and co-authorship (**b**) networks. We compute five $\beta$ for each network and control the opinion flow from low- to high-status agents. The *green lines* in the plots correspond to *egalitarian* societies (100 % opinion flow), whereas the *red lines* represent the *stratified* societies (0 % opinion flow). The *lines* in between (*black*, *blue* and *magenta*) depict the *ranked* societies, in which the opinion flow from low- to high-status agents is inhibited to 75, 50 and 25 %, respectively. For readability reasons, *error bars* representing standard deviation of the mean agent’s inventory size over 100 runs per $\beta$ are not depicted in the plots. In the English StackExchange network (**a**), in the case of an *egalitarian* society a common opinion is reached and the convergence rate is fast. In a *stratified* society, the opinions do not converge (the mean number of opinions lies between 1 and 2). *Ranked* societies also reach a common opinion with the highest convergence rate. Thus, for the English network, the consensus building depends on the status but in a non-obvious way, indicating that there is a specific setting at which the influence of the social status reaches the optimal state. In the case of co-authorship network in **b**, consensus is reached almost independently from $\beta$, so external interventions (such as our Probabilistic Meeting Rule) do not influence opinion convergence rates (color figure online)

Figure 4 summarizes the results of our experiments by depicting the agent’s inventory size as a function of the simulation progress for the (a) English StackExchange and (b) co-authorship networks.

### Inventory size evolution of disassortative networks

The simulation results among all StackExchange networks are similar; thus, we show only the results of the largest StackExchange network (i.e., English in Fig. 4a). In the case of *egalitarian* society ($\beta =0$), the English network converges to a single opinion. This is in line with the previous experiments with the Naming Game—in networks without a strong community structure, we always reach a consensus. In the case of *stratified* society, we do not observe convergence—consensus cannot be reached. This seems slightly counterintuitive—an intuition would be that consensus building would benefit from the presence of agents with a high social status and their influence on agents with a lower social status.
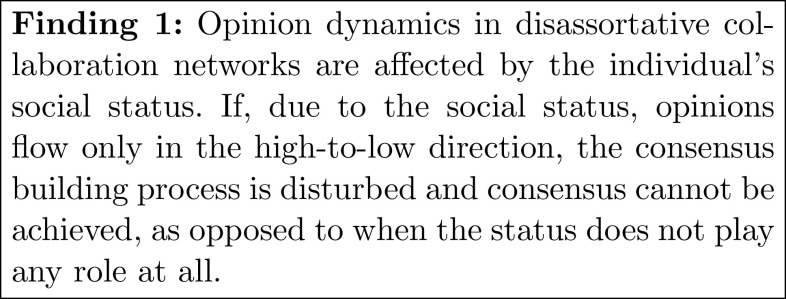


The simulation results for *ranked* societies indicate that the impact of the social status on opinion dynamics is a complex one. In all our StackExchange networks, we observe the following situation. By starting at $\beta =0$ and slowly increasing the stratification factor (note that higher values of stratification factor successively reduce percentages of meetings between low- and high-status agents), we are at first still able to reach consensus. Moreover, the convergence rate increases with a slightly increased stratification factor (cf. Fig. 4a for e.g., *ranked* 75 %—black line with triangle marker and *ranked* 50 %—blue line with diamond marker). However, by further increasing the stratification factor, we reach a tipping point after which a further increase of the stratification factor results firstly in slower convergence rates before we again reach a state of no convergence at all (within e.g., *stratified* society).
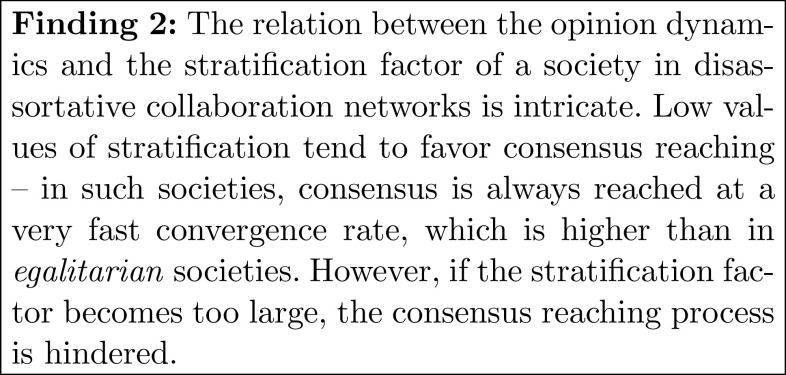


### Inventory size evolution of assortative networks

Due to the large size of the co-authorship network, a much higher number of interactions are needed in order for all agent pairs to participate at least once in a meeting. For our experiments, we used 20 million interactions, but if the number of interactions is further increased the lines in Fig. 4b will continue to drop toward 1. The co-authorship network is characterized with a positive assortativity coefficient that indicates that high-status agents are, on average, connected to other high-status agents, and low-status agents are connected to other low-status agents. The number of connections between low- and high-status agents is low; therefore, few meetings are taking place between these two classes. Consensus is reached almost independently from $\beta$ (cf. Fig. 4b), so our Probabilistic Meeting Rule does not benefit faster opinion convergence rates.
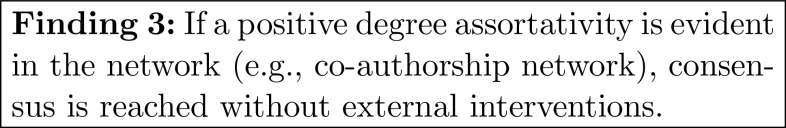


### Participation of agents in meetings across status groups

To further analyze these findings, let us investigate in more details the direction and intensity of opinions flow in our disassortative and assortative networks. To that end, we separate the agents into two classes: high (agents with the status above 90th percentile) and low (agents below 90th percentile) class. All reputation distributions are skewed to right and resemble a heterogenous distribution, and the division into classes results in a reputation boundary of for example, 220 for English StackExchange network with all agents having reputation above 220 belonging to the high class and all agents below 220 belonging to the low class (for comparison the highest reputation score in English dataset is 105,678). All other StackExchange networks are comparable to English, and our analysis produces similar results. For that reason, we henceforth discuss only the English network as an example of our disassortative networks. In the case of our assortative network (i.e., co-authorship network), the highest reputation score is 15,758 and the reputation boundary for the 90th percentile is at 27, indicating that all low-status agents have a reputation score below 27, while high-status agents possess a reputation score above 27.

**Fig. 5:**
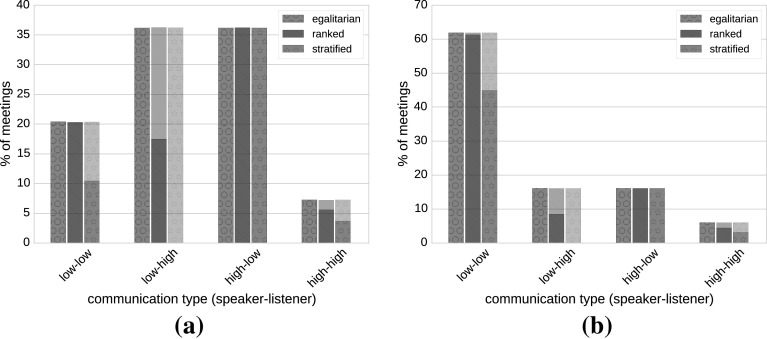
Participation of agents in meetings across status groups. The percentage of interactions resulting in meetings as a function of reputation classes in the English StackExchange (**a**) and co-authorship network (**b**). The high class comprises agents with the status above 90th percentile and the low class all other agents. In the *stratified* society (*red bars *with star texture), a common opinion cannot be reached because the meeting rule is so strict that even communications between low agents (low-to-low pairs) are severely impaired. In the *egalitarian* society (*green bars* with *circle* texture), the convergence is slower because low-status agents disturb high-status agents by inflicting their opinion upon them (low-to-high pairs). In the *ranked* society (*blue bars* with *line* texture), the optimal convergence is achieved because low-status agents can diffuse opinions among themselves (low-to-low pairs). At the same time, since the communications between low- and high-status agents are inhibited (low-to-high pairs), low-status agents’ opinions cannot disturb those of high-status agents. The only difference between the plots in **a** and **b** lies on the percentage of meetings among low-status agents and between low- and high-status agents. Since in the co-authorship (assortative) network, agents belonging to the similar classes tend to connect together, the number of meetings between low-to-low pairs is higher than the number of meetings between low-to-high and high-to-low pairs (color figure online)

An important question is what happens when agents interact and how the Probabilistic Meeting Rule evaluates depending on the classes of agents participating in a meeting. In other words, we want to investigate the fraction of interactions that turn into a successful meeting (which consequently results in an opinion flow and increases the likelihood of two agents agreeing on a single word). We therefore classify each interaction according to the agent classes into four possible pairs: (i) low-to-low, (ii) low-to-high, (iii) high-to-low and (iv) high-to-high where the first class corresponds to the speaker’s class and the second corresponds to the listener class. Figure 5 depicts the fractions of successful meetings among all interactions in the English StackExchange and co-authorship networks for three values of the stratification factor—*egalitarian* society (corresponds to $\beta =0$), *ranked* society (up to 50 % opinion flow is allowed between low- and high-status agents with optimal values $\beta =0.0001$ for English and $\beta =0.005$ for co-authorship network) and *stratified* society (e.g., $\beta =1$ and $\beta =5$). The only difference between plots in (a) English (disassortative) and (b) co-authorship (assortative) networks lies on the percentage of meetings taking place among low-status agents and between low and high agents. As previously mentioned, the number of physical connections between low and high agents in the co-authorship network is lower than in StackExchange networks, and this results to the lower number of meetings taking place between these two classes. Since, in the co-authorship network agents belonging to the same classes tend to connect together, the number of meetings among low agents (low-to-low pairs) is much higher compared with StackExchange networks. The fraction of high-status agents is equivalent for both networks; thus, the number of meetings taking place between high-status agents is almost the same.

In the case of *stratified* society (red bars with star texture), opinions flow without restrictions only in high-to-low direction. Thus, the agents with a higher status can pass over their opinions to the agents with a lower status. The flow in the opposite direction is completely prohibited, and therefore, agents with a lower status cannot influence the opinions of the agents with a higher status. However, the Probabilistic Meeting Rule in this case is so strict and prohibitive that it greatly inhibits the opinion flow within the agents of the same status (i.e., high-to-high and low-to-low pairs). Because of the skewed nature of the reputation distributions, the inhibition in the low-to-low group (which is considerably larger than the high–high group) is more severe—the agents with a lower social status cannot efficiently exchange their opinions with each other and must rely on the agents with a higher social status to inject opinions into the low group by meeting each low agent separately. Since there are few high-status and many low-status agents, consensus is never reached.

On the other hand, in the case of *egalitarian* society (green bars with circle texture), opinions flow without any restrictions in all directions. This results in the convergence of opinions and a rather fast convergence rate. However, the convergence rate is slightly slower as compared to the optimal case (*ranked* society). In our opinion, the explanation for this phenomenon lies in the dynamics of the low-to-high group meetings. Since everybody can impose her opinion onto everybody else, low-status agents very often change the opinions of high-status agents. Thus, low-status agents increase the variance in the inventories of high-status agents, and they need additional meetings to eliminate these opinions. This results in slower convergence rates.

A particular dynamics of low-to-high meetings also explains faster convergence rates in *ranked* societies (blue bars with line texture). In this case, the opinion flow from the agents of low status to the agents of high status is strongly slowed down. Therefore, the disturbances in the opinions of high-status agents are not substantial any more. On the other hand, as opposed to the *stratified* society, the opinion flow within the low-to-low group is not impaired at all. Thus, the injected opinions from the high-status agents can be diffused among the low-status agents themselves without need to address each low-status agent separately. This, combined with the reduced disturbances flowing from low- to high-status agents, results in optimal opinion convergence rates.
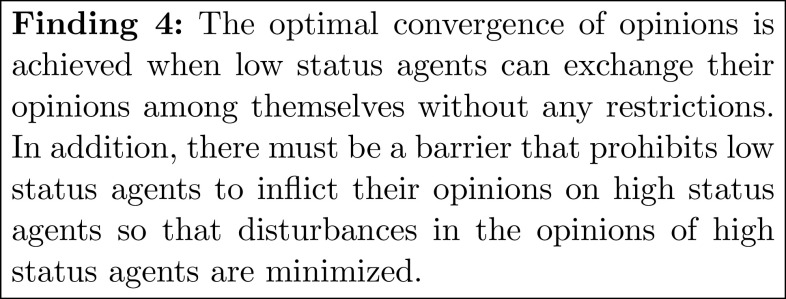


### Agents’ final agreement

In order to gain insights into the final agreement of individuals, we investigated each of the single opinions that agents agreed on. So, we modified the initialization of the agents’ inventories to differentiate between opinions assigned to low- and high-status agents, respectively. After rerunning the experiments and evaluating the results, we found out that for very low stratification factor  (correspond to higher percentages of meetings taking place between low- and high-status agents, e.g., *egalitarian*, *ranked* 75 % and *ranked* 50 % in Fig. 4) the final agreement of agents is mostly on the opinion of a low-status agent, whereas for higher stratification factor (e.g., *ranked* 25 % and *stratified* in Fig. 4) the opinion on which all agents agreed on is usually one of a high-status agent. This is in line with the fact that for very low stratification factor the intensity of the communication from low- to high-status agents is high, so the probability that an opinion of a low-status agent is the final opinion on which all agents agreed on is high. By increasing beta, we decrease the probability of a communication taking place between low and high agents. Thus, the final agreement is mostly on the opinion of a high-status agent.
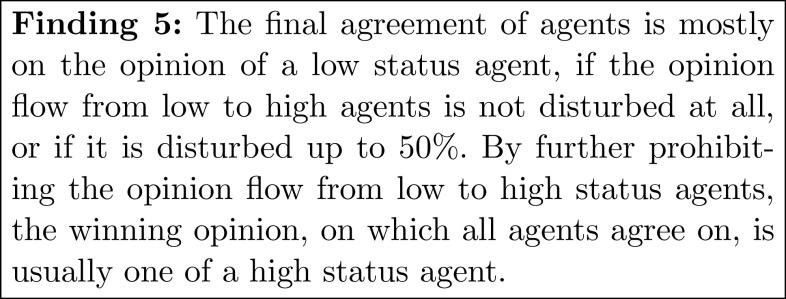


## Analysis of network correlations

In this section, we study how network structure and, in particular, the correlation of structure and status affect the process of consensus reaching in collaboration networks by constructing disassortative and assortative synthetic networks.

### Decorrelating networks

Our aim is to study in detail how the network structure and, in particular, the correlation of structure and status affect the process of consensus reaching in our networks. Obviously, the connections between hubs and other nodes play a crucial role, as well as the distribution of degree sequence and the position of high-reputation nodes in the network. For this study, we generated specific synthetic networks, whereas in each case, only one particular property of interest is preserved while others are eliminated. This way, in each experiment, we can assess the influence of a single property on the overall opinion dynamics process.

#### Degree and status correlation

In order to analyze the role of network structure and especially the role of the degree assortativity on the process of opinion spreading, we generate three synthetic networks based on the original collaboration networks introduced in Sect. 3. All synthetic networks have the same number of nodes *n* and edges *m* as the empirical networks, but we modify the connections between nodes and the correlation between degree and reputation as follows:

*Random network*: Here, we rewire the edges uniformly at random. This means that all nodes have equal probability of getting selected for creating an edge. The resulting network corresponds to the Erdős–Rényi model proposed in Erdös and Rényi (1959), and its node degree distribution follows a homogeneous Poisson distribution. With this network, we eliminate the degree sequence and the correlation with reputations.
*Configuration model*: In this case, the edges from the original network are randomly rewired, but the degree sequence remains the same (Bender and Canfield 1978; Molloy and Reed 1995). An uncorrelated rewiring minimizes the bias for connections in a network as all nodes are randomly rewired to different nodes than in the original network. Since the degree sequence is not modified, this results in a heterogeneous degree distribution with the same slope as in the original network. With this network, we eliminate the correlation between nodes over the edges, for example, we eliminate the correlations caused by the friendship relations.
*Shuffled reputations*: Finally, we do not modify the network structure itself, but shuffle the reputation of nodes randomly. In the resulting network, the node degrees are decorrelated with reputations.

For all the experiments in the synthetic networks, we use as basis the English StackExchange and the co-authorship datasets and we follow the experimental setup described in Sect. 3.

### Results of decorrelated networks

**Fig. 6:**
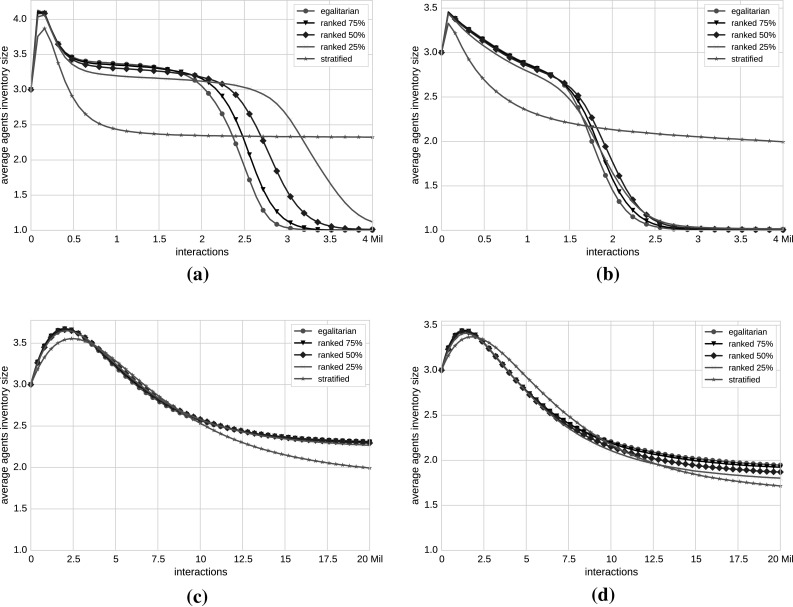
Decorrelating networks. Mean values of the agent’s inventory size in relation to the number of interactions for English Erdős–Rényi (**a**), English shuffled reputations (**b**), co-authorship Erdős–Rényi (**c**) and co-authorship configuration model (**d**) networks. The process of consensus building varies among networks. In the English Erdős–Rényi network, the process of consensus reaching is slowed down, whereas in the English shuffled reputations, the opinion convergence rate is faster (agents agree to a common opinion almost independently from $\beta$). In the English configuration model, opinions converge with the highest rates in the case of *ranked* societies (e.g., *ranked* 50 %), which corresponds to the English original network; thus, the plot is omitted. In the co-authorship Erdős–Rényi and configuration model, the consensus building process is slowed down compared with the co-authorship original network. The simulation results of the co-authorship shuffled reputations network are identical with the original co-authorship network; consequently, it is not included in the figure

Our experimental results reveal some interesting insights. In Fig. 6, we show the evolution of agent’s inventory size during the interactions, averaged over 100 runs. To better understand the variation of the stochastic processes performed throughout our simulations, we calculated standard deviations over 100 runs per $\beta$, but for readability reasons, we removed error bars from the plots. Typical standard deviation values range between 0.48 (e.g., English shuffled reputations network) and 0.66 (English Erdős–Rényi network). Table 2 summarizes the results of our experiments both on empirical and decorrelated networks.

#### Disassortative networks

We recall the results of the original English network once more for an easier comparison with the results with synthetic networks. The simulation results with the English StackExchange original networks show that the *ranked* societies reach a common opinion with the highest convergence rate, higher than in *egalitarian* societies (e.g., *ranked* 50 % compared to *egalitarian* in Fig. 4), whereas in a *stratified* society consensus is not reached at all.

The simulation results for the English Erdős–Rényi network differ from the original network (see Fig. 6a). Except for *stratified* society, for which consensus is not reached within the limit of interactions, for other societies, the process of consensus reaching is slowed down. The fastest convergence is achieved with $\beta = 0$, respectively, in *egalitarian* societies. This result shows that the convergence rate is highly dependent on the existence of hubs in a network. In an Erdős–Rényi network, the high-status agents are not hubs any more since their degrees are much smaller and therefore they cannot spread their opinions to low-status agents as quickly as in the original network.

We find a further evidence for this behavior in the English configuration model in which the calculated stratification factors and the evolution of agent’s inventory size are identical to the original network; thus, the figure presenting the results is not included. In this example, we keep the same degree sequence but rewire the edges in the English StackExchange network. Since we now keep the hubs and the degree–status correlation, we do not disturb the consensus reaching process. We simply reconnect the low-degree/low-status agents to different high-degree/high-status agents. This result also shows that additional external correlations such as friendship/collaboration correlations do not influence the consensus reaching. Mainly, it is the degree/status correlation that provides support for achieving the consensus.

In the English network with shuffled reputations (Fig. 6b), the estimated stratification factors that define the five societies are identical to the English Erdős–Rényi network. However, agents agree to a common opinion almost independently from the society form, except for the *stratified* society. The convergence rate is faster than in English Erdős–Rényi network. This outcome indicates that in networks with heterogenous degree distribution and uncorrelated reputations of users, consensus is reached automatically without need for external interventions. Since, however, in most of empirical collaboration networks degree strongly correlates with user reputation, we need another mechanism that can positively influence opinion dynamics. That mechanism includes controlling the communication between low- and high-status nodes through the stratification factor.

**Table 2:** Summary of our findings

Network	Type	*Egalitarian*	*Ranked* 75 %	*Ranked* 50 %	*Ranked* 25 %	*Stratified*
Disassortative English StackExch.	Empirical	Converge	Converge	Fastest convergence	No converge	No converge
	Erdős–Rényi	Fastest convergence	Slowed down	Slowed down	Slowed down	no converge
	Configuration model	Converge	Converge	Fastest convergence	No converge	No converge
	Shuffled reputations	Converge	Converge	Converge	Converge	No converge
Assortative co-authorship	Empirical	Converge	Converge	Converge	Converge	Converge
	Erdős–Rényi	Slowed down	Slowed down	Slowed down	Slowed down	Slowed down
	Configuration model	Slowed down	Slowed down	Slowed down	Slowed down	Slowed down
	Shuffled reputations	Converge	Converge	Converge	Converge	Converge

Table summarizing the results of our work

#### Assortative networks

Figure 6c shows the simulation results of the co-authorship Erdős–Rényi network, in which the hubs are removed. The calculated $\beta$ differs from the empirical co-authorship network and the consensus reaching process is slowed down in this case. This outcome confirms once more that the presence of hubs is crucial for the consensus reaching process.

Applying the configuration model to the co-authorship network while keeping the degree sequence changes the connection patterns between nodes. So, rewiring the edges reduces the number of high-to-high and low-to-low connections, simultaneously increasing the number of high-to-low links. This results in a decreased assortativity. In fact, in the configuration model, we measure the assortativity coefficient of 0.0001, whereas in the original co-authorship network that factor is 0.15. This is shown also in Fig. 6d, where the opinion convergence rates are slowed down.

Shuffling the reputations in the co-authorship network does not impact the simulation results as they are identical with the empirical co-authorship network. Thus, the respective plot is omitted from Fig. 6.

#### Distribution of status differences

**Fig. 7:**
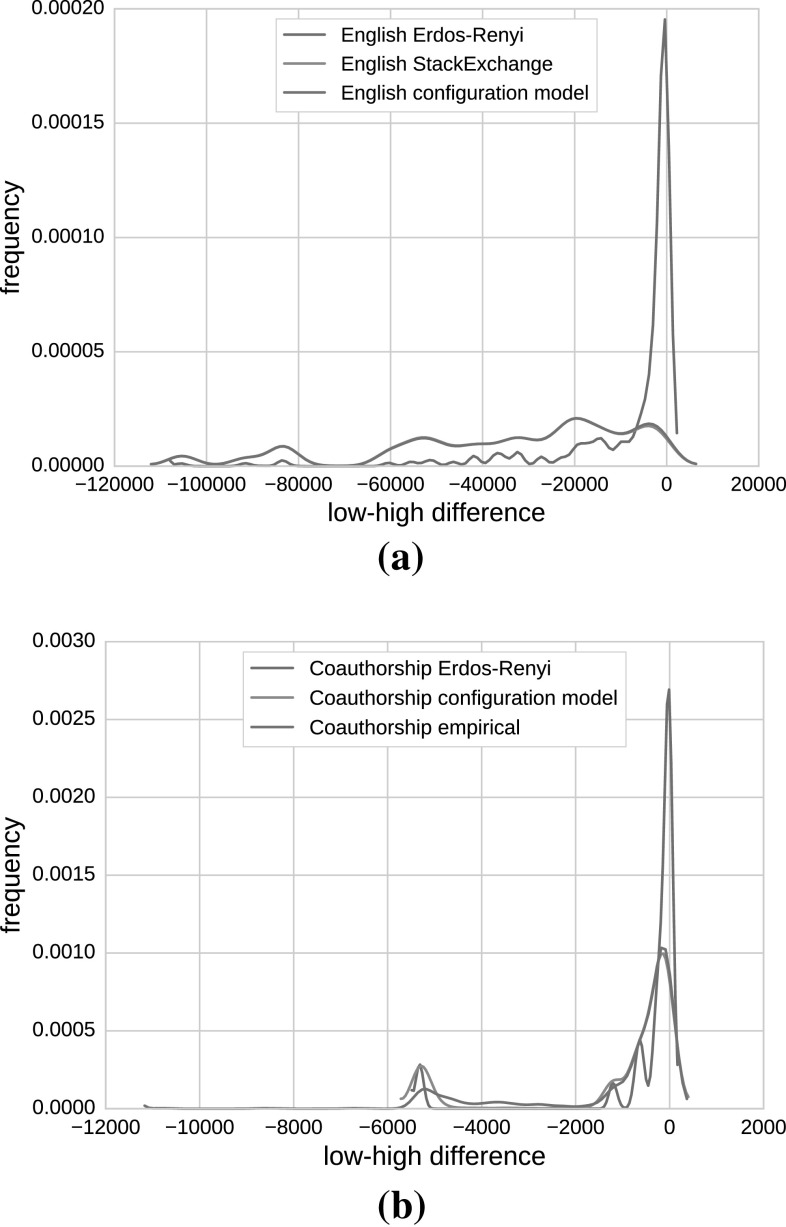
Kernel density estimation of the distribution of status differences between low and high agents. Disassortative networks are shown in **a** and assortative networks in **b**. The distribution of agents’ status differences in the English StackExchange and configuration model networks in **a** are almost identical; thus, the *blue* and the *red lines* overlap. Due to many connections from low- to high-status agents, we frequently see high negative differences. In the English Erdős–Rényi network (*blue line*), the majority of differences between low- and high-status agents is close to 0, because of the lower number of connections between these two groups of agents. The English shuffled reputations network is not shown in the plot, because of very-low-status differences with only one peak around 0. In **b** are shown lower differences between agents’ statuses in the co-authorship empirical network and synthetic networks (color figure online)

To further quantify our findings, we investigated the distribution of status differences between two connected nodes in our networks. The differences are calculated for two neighboring nodes if one of the nodes is a low and the other one is a high-status node (defined by the 90th percentile). The results for disassortative and assortative networks are depicted in Fig. 7.

In the networks with a heterogenous degree distribution, a negative degree assortativity and a strong correlation between degree and status (red and green lines in Fig. 7a), there are many connections from low- to high-status nodes and therefore we frequently observe high negative differences. In other words, there are many potential meetings between low and high agents that given that they take place often can disturb the high-status agents and consequently the consensus reaching process. Thus, to reduce the number of meetings that take place we need to apply a mechanism such as our Probabilistic Meeting Rule and inhibit the opinion flow in the low-to-high direction.

In the case of the English Erdős–Rényi network (blue line in Fig. 7a), there are lower differences between low- and high-status agents (the majority of differences is close to 0), due to the lower number of connections between these two groups of agents. Thus, not many of the meetings that take place are high-to-low agent meetings and additionally with our Probabilistic Meeting Rule, we are also prohibiting the opinion flow from low- to low-status agents. Consequently, this slows down the consensus reaching process.

In the English shuffled reputation network, the number of connections between low- and high-status agents is the same as in the original network, but the differences between agents’ statuses are lower (with only one peak close to 0, thus, it is omitted in Fig. 7a), which speeds up the consensus reaching even without external interventions such as Probabilistic Meeting Rule.

In Fig. 7b, it is shown that, in general, there are lower differences between agents’ statuses in the co-authorship empirical network and synthetic networks derived from it, which explains the fact that in co-authorship original network consensus is reached fast and independent from $\beta$. The opinion convergence rates are slowed down only if the presence of hubs is lower or if the degree assortativity is decreased.
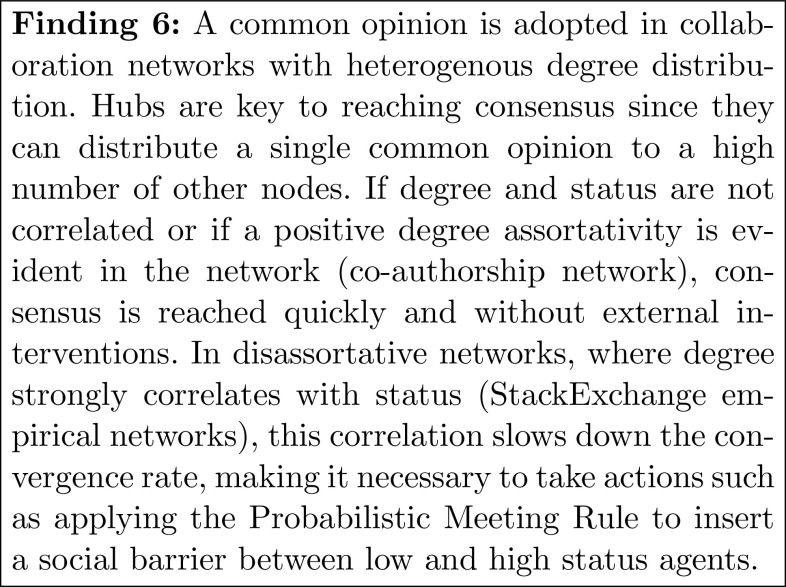


## Related work

At present, we identify three main lines of research related to our work: opinion dynamics, social status theory and naming game.

### Opinion dynamics

Opinion dynamics is a process characterized with a group of individuals reaching a consensus (i.e., the majority of a group share the same opinion). In opinion dynamics, the focus is on modeling the opinion state of an individual in particular and a population in general. Opinion dynamics has been tackled in the past in the context of statistical physics (Castellano et al. 2009; Iniguez et al. 2014). As discussed in Castellano et al. (2009), if opinion dynamics is viewed from a perspective of statistical physics, an individual is analogous to a particle with properties that may or may not change over a period of time. Thus, the social process of interaction among individuals can be designed as a mathematical model that represents a change in the local and global state of an individual and a group. One of the examples of such a process is the Naming Game model, a variant of which we are using in our work, that models how individuals behave during a meeting and exchange their opinions. In our experiments, the meeting process is further enhanced by taking reputation scores of individuals into account. Constraining the system to favor high-reputation nodes resulted in reaching consensus later as compared to an unconstrained model.

In a different context opinion, dynamics is studied in Blondel et al. (2010), Hegselmann and Krause (2002), Krause (2011), Lorenz (2007) and Muller (2006), where an opinion is represented as a real number and a classical approach of individual opinion formation involves averaging over opinions of other agents in the system. In such a setting, a consensus is considered to be reached if all the agents in the system agree to the same value of opinion. The process of opinion dynamics is studied in Krause (2011) from both the local and global perspective. They defined the opinion formation process as local when a user takes into account only the nearest neighbors, whereas in the case of global opinion formation the user takes into account all other agents in the network. The process of opinion formation is studied in Blondel et al. (2010) by means of a continuous time multi-agent system. In their work, they proved that opinion converge to a set of clusters, where agents in each of the cluster share a common value. Lorenz (2007) studied a continuous model of opinion dynamics under bounded condition. The bounded condition restricts users to interact with their peers only if they are close to each other. Such a process of opinion dynamics leads to formation of clusters with characteristic location and size patterns. They found the drifting phenomenon in composition of cluster in case of heterogeneous bounds. Muller (2006) studied the process of internal organization within communities of practice and how such a process leads to some members obtaining a leadership status. They developed a model to depict the self-organizing process and found that leaders are the members who correspond to higher level of activity in the community.

### Social status theory

Research on how the position and status of a node influence a network is mostly carried out in the context of network exchange theory (Markovsky et al. 1993; Walker et al. 2000; Willer 1999). This theory states that connections and a position in a network lead to a power condition that is based on how the nodes are connected and which position they take in the network (Walker et al. 2000). For example, in Markovsky et al. (1993), researchers differentiate between weak and strong powers network in terms of node positions and network properties. The authors give a theoretical extension to the network exchange theory to explain why in sparsely connected networks a stronger power effect is observed than in densely connected networks. They found that in densely connected networks, weak position nodes have an advantage since they have a higher connectivity, which enables them to short circuit the structural advantages of strong position nodes. This is related to our work, as we concentrate on investigating how the reputation of a node in a network affects the spread of opinion that leads to establishing consensus in the network. Also, we define various classes of nodes based on reputation and determined how their interaction affects their overall process of consensus building.

### Naming Game

The Naming Game has been introduced in the context of linguistics (Dall’Asta et al. 2006b) and the emergence of a shared vocabulary among agents (Baronchelli et al. 2006b) with the aim to demonstrate how autonomous agents can achieve a global agreement through pairwise communications without central coordination (Zhang et al. 2014). With that regard, we present a selection of variations of the Naming Game that are relevant to our work.

Similarly to our approach, the work of Brigatti (2008) describes a variation of the Naming Game that incorporates the agents’ reputation scores. In the beginning, reputation is randomly distributed (Gaussian distribution) among the agents. Successful communication increases the agents’ reputation, and during each iteration, the agent with a higher reputation score acts as a teacher and the one with the lower score as a learner. The main difference from our work is that in Brigatti (2008), they use synthetic data for the simulations and that the assigned reputation scores are random numbers that change during iterations. In our work, we employed empirical collaboration networks from StackExchange with reputation scores that were assigned by the community. As opposed to the work of Brigatti (2008) where there is an open-ended game with unlimited number of words, the inventory of our agents consists of predefined sets of three opinions.

Other examples for the Naming Game variations include the works of Liu et al. (2011), who studied the impact of spatial structures (e.g., geographical distances) have on meetings between individuals in a network, and Yang et al. (2008), who proposed a Naming Game that follows an asymmetric negotiation strategy and investigated the influence of hub effects on the agreement dynamics with specific focus on how quickly consensus could be achieved. Each agent in the network is assigned a weight defined by the agent’s degree and a tuneable parameter $\alpha$. During iteration, two nodes are randomly selected and based on their degree and the configuration of the parameter $\alpha$, they are either the speaker or the listener (i.e., if $\alpha > 0$, high-degree agents have more chances to be speakers and vice versa). This way, the dynamics of the game can be investigated in light of the varying influence of high-degree agents. Our work is somewhat related as we also use a parameterized probability function to define the probability of a meeting taking place between two nodes, in our case depending on their reputation score. The main difference to our work is that agents’ selection is unbiased and empirical data with explicitly provided reputation scores are used.

The diffusion of opinions across networks and the potential of reaching consensus are strongly influenced by the availability of communities and, specifically, by the presence of strong community boundaries (Lu et al. 2009). To investigate this effect, Lu et al. (2009) assigned a group of nodes in a network as a committed fraction, that is, nodes that are not influenced by other nodes in a network and do not ever change their opinion. In our dataset, however, no strong community structures are present.

## Conclusion and future work

Understanding opinion dynamics and how consensus is reached in social networks has been an open and complex challenge in our community for years. In this work, we addressed a subproblem related to this challenge by investigating a specific case of collaboration networks in which individual nodes have a certain social status.

To that end, we presented an extension (Probabilistic Meeting Rule) to the standard Naming Game model of opinion dynamics. We evaluated our approach on six large empirical collaboration networks, as well as on three specifically created synthetic networks, which reflected the characteristics of the empiric networks. In this work, we provided a computational approach for the general estimation of the stratification factor of our Probabilistic Meeting Rule and we analyzed the role network structure plays in the process of consensus building. These studies constitute the methodological contribution of our work to the field of opinion dynamics. Additionally, we investigated various real-world scenarios such as the emergence and disappearance of social classes in collaboration networks. From the empirical point of view, our investigations revealed insights about the influence of social status on the diffusion of opinions. Our main finding indicates that social status strongly influences the opinion dynamics in a complex and intricate way. More specifically, weakly stratified societies reach consensus at the highest convergence rate, whereas completely stratified societies do not reach consensus at all. The most important issue in this process is related to low-status agents and how their communication is controlled. In particular, the optimal convergence is achieved when (i) low-status agents are allowed to freely exchange opinions between themselves (since this reduces the need for high-status agents to interact with low-status agents) and (ii) simultaneously there is a communication barrier reducing the number of interactions of low-status agents toward high-status agents (since this reduces the variance in opinions of high-status agents). Furthermore, our investigations on the role of the network structure reveal that hubs are in general crucial to reach consensus, since they can spread a single common opinion to a high number of nodes. In assortative networks, in which connections between low and high agents are very rare, external interventions do not benefit faster convergence rates. A similar situation is observed in disassortative networks when degree is not correlated with a user’s status. If there is a strong correlation between status and degree in a disassortative network, this slows down the convergence rate, making it necessary to take actions such as applying the Probabilistic Meeting Rule to disturb the communication between low- and high-status users.

### Limitations

In our opinion, our work has the following limitations. Firstly, we represent social status with a single number—for certain scenarios this representation may be too simplistic. For example, people often play different roles in social networks and a non-simple interplay between the roles and status may exist. Secondly, a more finely grained classification of agents into various groups (e.g., low, mid and high groups or even finer divisions) may shed more light on the opinion dynamics. Finally, in our work, we consider only static snapshots of networks and reputation scores. However, not only opinions but also networks are dynamic, as new agents may arrive to the network, new edges may form and inactive edges may disappear from the network. Moreover, reputation itself is very dynamic and depends on the agent’s activity and the current perception of an agent by her peers.

### Future work

In our future work, we plan to address some of the limitations of our current work and extend our approach and experiments to other scenarios. For example, one interesting avenue for further research are the networks with a strong community structure. As communities tend to slow down the consensus reaching process, it would be interesting to investigate how status and/or network structure can be adjusted to support the process. Apart from social status, the influence of trust is of utmost importance in various social systems and in particular in social media. Thus, adapting the presented approach to analyzing how trust relates to opinion dynamics is another promising research direction for the future.

## Footnotes

1
http://stackexchange.com/.

2
https://aminer.org/DBLP_Citation.

3
http://faculty.nps.edu/volpano/.

4
https://github.com/floriangeigl/reputation_networks.
